# Circ_ZNF778_006 promoted ESCC progression by upregulating HIF-1α expression via sponging miR-18b-5p

**DOI:** 10.1038/s41598-023-46832-3

**Published:** 2023-11-08

**Authors:** Xianzhe Si, Xincheng Su, Weijie Lin, Jie Xu, Wenbo Huang, Feng Chen, Zhijun Huang, Jianqing Lin, Zhiyao Chen

**Affiliations:** 1 Department of Gastrointestinal and Esophageal Surgery, The 2nd Affiliated Hospital of Fujian Medical University, Quanzhou, 362000 Fujian Province China; 2 Department of Gastrointestinal Surgery, The Union Hospital of Fujian Medical University, Fuzhou, 350000 Fujian Province China; 3 Department of Oncology, The 2nd Affiliated Hospital of Fujian Medical University, Quanzhou, 362000 Fujian Province China

**Keywords:** Cancer, Oncology

## Abstract

In multiple malignant tumors, circular RNAs (circRNAs) are believed to play a crucial role. Our prior results demonstrated that circ_ZNF778_006 was significantly increased in esophageal squamous cell carcinoma (ESCC) tissues, but the roles of circ_ZNF778_006 in ESCC is still not clear. The expression of circ_ZNF778_006 was compared in different pathological grades of ESCC. And the expression levels of circ_ZNF778_006, miR-18b-5p, HIF-1α were analyzed by qRT-PCR and Western blot, respectively. Plasmid transfection techniques were applied to prepare ESCC cells with silenced or overexpressed genes (CircZNF778_006, miR-18b-5p). The CCK8 kit was used to determine cell proliferation, and the Transwell assay was used to measure the migration and invasion. The effects of circ_ZNF778_006 on tumor growth was investigated in vivo. Furthermore, luciferase reporter gene assay and RNA-binding protein immunoprecipitation (RIP) were performed to verify the targeting relationship between miR-18b-5p and circZNF778_006, miR-18b-5p and HIF-1α. The expression of circ_ZNF778_006 was positively correlated with pathological grade in ESCC. Circ_ZNF778_006 significantly inhibited sensitivity to 5-fluorouracil & cisplatin. It could promote the proliferation, invasion, migration in ESCC cells and accelerated tumor growth in vivo. Furthermore, circ_ZNF778_006 could upregulate the expression of HIF-1α via sponing miR-18b-5p. Circ_ZNF778_006 promoted ESCC progression by upregulating HIF-1α expression via sponging miR-18b-5p.

## Introduction

Esophageal cancer (EC) is one of the most common malignant tumors in the world. According to the epidemiological survey conducted by the World Health Organization (WHO) in 2020, the morbidity and mortality of EC ranked seventh and sixth, respectively. Squamous cell carcinoma accounts for more than 90% of EC in parts of Asia and sub-Saharan Africa1^1^. And the 5-year overall survival rate of EC is still poor, although combing multiple therapies improves the effects of treatment for advanced EC^2^. Therefore, the elucidation to EC pathogenesis may be particularly important to the understanding of cancer pathogenesis.

Circular RNA (circRNA) is a unique class of endogenous non-coding RNA that forms a circular structure by connecting the 3′ end to the 5′ end of the RNA^3^. It exist in eukaryotic cells that play an important role in many biological processes, especially cancer process^4^. In the last several years, circRNAs have also been reported to be associated with ESCC. Zhou et al. found that circ_0000277 promotes the occurrence and metastasis of ESCC cell tumors via promoting the expression of LAMA1 through miR-4766-5p^5^. Chen et al. found the level of circNTRK2 increase significantly in ESCC and promoted cell proliferation and invasion^6^. Our prior sequence data showed that the circ_ZNF778_006 is significantly increased in ESCC (Suppl1). Although the role of circ_ZNF778_006 in cancer pathogenesis is still not studied.

MicroRNAs (miRNAs), a non-coding RNA of approximately 22 nucleotides, can regulate gene expression by complementary pairing with target genes. It played an important role in tumorigenesis and development^7^. As a non-coding RNA, MiR-18b-5p plays a regulatory role in multiple tumors. Jin et al.^8^ found that miR-18b-5p can inhibit the proliferation of gallbladder carcinoma. And Xu et al.^9^ found that the combination of miR-18b-5p and circ-FBXW7 can improve the resistance to oxaliplatin in colorectal cancer. However, the regulatory role of miR-18b-5p in ESCC is still not clear.

HIF-1α is a ubiquitous nuclear transcription factor in hypoxic tissues. It’s a key oxygen-dependent regulatory factor for cells to respond to hypoxia signals. And it play an important role in the development of malignant tumors by mediating angiogenesis, cell metabolism, cell invasion and migration, and apoptosis^10,11^. Zhao et al.^12^ found that HIF-1α is a survival factor for the prognosis of ESCC. Du et al.^13^ found that silent SNHG6 can inhibit the progression of ESCC through the miR-186-5p/HIF1α axis. Although, the role of the network of circ_ZNF778_006/ miR-18b-5p/HIF1α in progression of ESCC remains undifined.

So our studies was to detect the role of the network of circ_ZNF778_006/ miR-18b-5p/HIF1α in progression of ESCC by in vivo and in vitro experiments.

## Materials and methods

### Clinical samples and cell culture

Tumor tissues and adjacent normal tissues were collected from 120 patients undergoing primary surgical treatment in the department of gastrointestinal and esophageal surgery in the 2nd affiliated hospital of Fujian medical university (FMU) from June 2020 to October 2021. Written informed consent was obtained from all patients before operation. The research was conducted with the approval of the Ethics Committee of the 2nd affiliated hospital of FMU. All the tissues were preserved at – 80 ℃ after freezing using liquid nitrogen. Meanwhile, the clinical consent was collected after surgery.

All cells were purchased from the Chinese biological medical cell research (BMCR) (Shanghai, China). Under a humidified atmosphere with 5%CO2 at 37 ℃, human normal esophageal squamous cell line (HEEC) and ESCC cell lines (ECA109, KYSE-150, EC9706, TE-1) were cultured in the Dulbecco’s modified Eagle’s medium (DMEM; Invitrogen, Carlsbad, CA, USA) with 10% fetal bovine serum (FBS; Invitrogen, CA, USA) and antibiotics (100 U/mL penicillin, 100 ug/mL streptomycin, KeyGen, Nanjing, China).

### Bioinformatic analysis

The location and length of circ_ZNF778_006 were found by using the circBank, the interact miRNAs of circRNAs were predicted by using Starbase software, and the potential target of miR-18b-5p was predicted by using the DIANA, miRDB, and TargetScan software.

### Real-time quantitative polymerase chain reaction(RT-PCR)

Total RNAs were extracted from tissues and cells with TRIzol(Takara, Dalian, China) according to the user’s manual. Reverse transcription was done according to the instructions in the ExScript TM RT Reagent Kit(Takara, Dalian, China). And the RT-PCR reaction was carried out on an ABI 7500 fast system(Foster City, CA, USA), and the fold changes were calculated by using the 2^−△△Ct^ method. The RT-PCR primers for circ_ZNF778_006 were designed based on the circPrimer2.0 software analysis. The control primer for circ_ZNF778_006/HIF-1α all and miR-18b-5p are GAPDH and U6, respectively. The Forward and Reverse primers are shown in Table 1.

**Table1:** sequences of primers for RT-PCR.

Names	Sequences(5’-3’)
circ_ZNF778_006: Forward	agacttcatcctgccctgtc
circ_ZNF778_006: Reverse	aggcctgagtgttcactgaa
miR-18b-5p: Forward	CGCGGATCCACCATGGTGATTTAATCAGA
miR-18b-5p: Reverse	CCGCTCGAGCCGTTCAAATCATTTCTCAA
HIF-1a: Forward	GAAACCACCTATGACCTGC
HIF-1a: Reverse	CTGTTTGTTGAAGGGAGAA
U6: Forward	CTCGCTTCGGCAGCACA
U6: Reverse	AACGCTTCACGAATTTGCGT
GAPDH: Forward	GTCAGCCGCATCTTCTTT
GAPDH: Reverse	CGCCCAATACGACCAAAT
Ki-67: forward	ACGCCT GGT TAC TAT CAA AAG G
Ki-67: Reverse	CAG ACC CAT TTA CTT GTG TTG A
E-cadherin: Forward	AAGACAAAGAAGGCAAGGT
E-cadherin: Reverse	AGAGAGTGTATGTGGCAATG
N-cadherin: Forward	TGAAGTCCCCAATGTCTCCA
N-cadherin: Reverse	GCATCATCATCCTGCTTATCC
Vimentin: Forward	CAGTCCTGGTGAATGATGT
Vimentin: Reverse	GTTAGTGCTCACCGATGCG

### Ribonuclease R(RNase R) and actinomycin D treatment assay

RNase R treatment: 2.5ug total RNA from ESCC cells was treated with RNase R(2U/mg, Epicentre, USA) for 30 min at 37 ℃, followed by extraction with Trizol reagent(Invitrogen, USA). And then the measurement of mRNA was performed using RT-PCR assay.

actinomycin D treatment: ESCC cells were seeded in a 6-well plate at a density of 1 × 10^5^ cells/well, and treated with actinomycin D(2ug/ml, Abcam, USA) for 0, 6, 12, 18, 24 h. Then the measurement of circ_ZNF778_006 and ZNF778 mRNA was performed using RT-PCR assay.

### Cell transfection

The sh-circRNA, miRNA inhibitor, miRNA mimic, siRNA, and their respective controls were provided by Ribobio Company(China). The cells were transfected with oligonucleotides (50 nM) and vectors (200 ng) at 37 ℃ based on the guidelines of the Lipofectamine 3000 reagent (Invitrogen, USA). All cells were collected for the next studies.

### Cell counting kit-8(CCK-8) assay

All cells were incubated for 1, 2, 3, 4, 5 days at 37 ℃, and then followed by incubated CCK-8(10uL) for another 4 h based on the guidelines of the CCK-8 kit(Dojindo, Japan). The assessment of absorbance at 450 nm was conducted based on the instruction of a microplate reader (Biotek, VT, USA).

### Luciferase activity assay

Cells were co-transfected with circ_ZNF778_006 plasmids or their mutant fragments and miR-18b-5p mimic or inhibitor by using lipofectamine 3000 reagents (Invitrogen, USA) according to the protocol. The luciferase activities were measured by using a dual-luciferase reporter assay kit after transfection for 48 h.

### Transwell assay

Cells in a serum-free medium were incubated in the upper chamber of the transwell. The cell culture medium containing fetal bovine serum was added to the lower chamber and incubated for 48 h. Then take out the cell culture medium, wash it twice with PBS, fix it with 4% paraformaldehyde for 15 min, and stain it with 0.1% crystal violet for 15 min. Before the cell invasion experiment, matrix gel was added to the upper chamber and incubated for 30 min at 37 ℃. Cell counts for each of the 5 visual fields were calculated by using the microscope camera (Nikon, Japan).

### RNA immunoprecipitation(RIP) assay

The RIP assay was undertaken in EC9706 and TE-1 cells with the EZ-Magna RIP™ RNA Binding Protein Immunoprecipitation Kit(Millipore, USA) following the protocol. The retrieved RNA was quantified using qRT-PCR as above described.

### Western-blot assay

Dissected cells were washed with PBS and total protein was extracted (10 mM Tris base- HCl, 150 mM NaCl, 1% NP-40, 1% Triton X-100, 5 mM EDTA, 0.1% SDS, 1% sodium deoxycholate, 1 mM phenylmethylsulfonyl fluoride, 1 mg/mL aprotinin, 1 mg/ml pepstatin, 1 mg/mL leupeptin, 1% sodium orthovanadate and 50 mM sodium fluoride). The concentration of lysates was determined by the bicinchoninic acid (BCA) assay. Lysates (80 mg) were separated in SDS–polyacrylamide gels and transferred to polyvinylidene fluoride (PVDF). Membranes were blocked with 5% non-fat dry milk and incubated with primary antibody HIF-1α (1:1000 dilution, Abcam, USA), followed by horseradish peroxidase-conjugated secondary antibodies. Immunoblots were developed with ECL (Millipore, Billerica, USA), according to the manufacturer’s instructions, and densitometric results were analyzed with Quantity One image-analysis software (Bio-Rad, CA, USA). Coefficients of variance were calculated by the ratio between means ± SE.

### In vivo investigations

The in vivo investigations using 20 Young male BALB/c nude mice (age, 6-week-old) mice were conducted strictly following the Institutional Animal Care and Use Committee (ICAUA) guidelines from the 2nd Affiliated Hospital of Fujian Medical University .The animals were raised under a 12-h light/dark cycle room in an SPF environment. To establish the tumor model, 20 Young male BALB/c nude mice (age, 6-week-old) were given an injection of ECA109 cells transfected with a normal vector or circ_ZNF778_006 vector (200 μL of 4 × 10^6^ cells/mouse) on the dorsal side of the animal. Further, the tumor volume was calculated using the equation, Volume = (Length × Width2)/2, in which length indicates the longest dimension while width represents the shortest dimension of the tumor. Finally, the mice were killed by cervical dislocation, and excised tumors were weighed accurately.

### Statistical analysis

Counting data were reported as a percentage (%), and quantitative data were reported as means ± standard deviation (SD). Two sample t-test was performed to compare the difference of values between two groups, and one-way ANOVA with a post-hoc Fisher’s least-significant-difference (LSD) test was applied to identify the significant change in value among several groups. A value of *P* < 0.05 was considered statistically significant. Statistical analysis was done by using SPSS version 23.0 for windows.

### Ethics approval and consent to participate

The protocol of this study was approved by the ethics committee of 2nd affiliated hospital of FMU, and written informed consent was obtained from each participant (approval no. FJMU-2020-099 and FJMU-2021-124). All the authors had access to patient information during data collection. This study was approved by the Ethics Committee of the 2nd Affiliated Hospital of Fujian Medical University.

All animal studies complied with relevant ethical regulations for animal testing and research. All samples were collected with informed written consent from the patients in strict accordance with institutional and legal ethical guidelines.

The study was carried out in compliance with the ARRIVE guidelines.

## Results

### The characters and the expression of circ_ZNF778_006 in ESCC tissues

**Figure 1:**
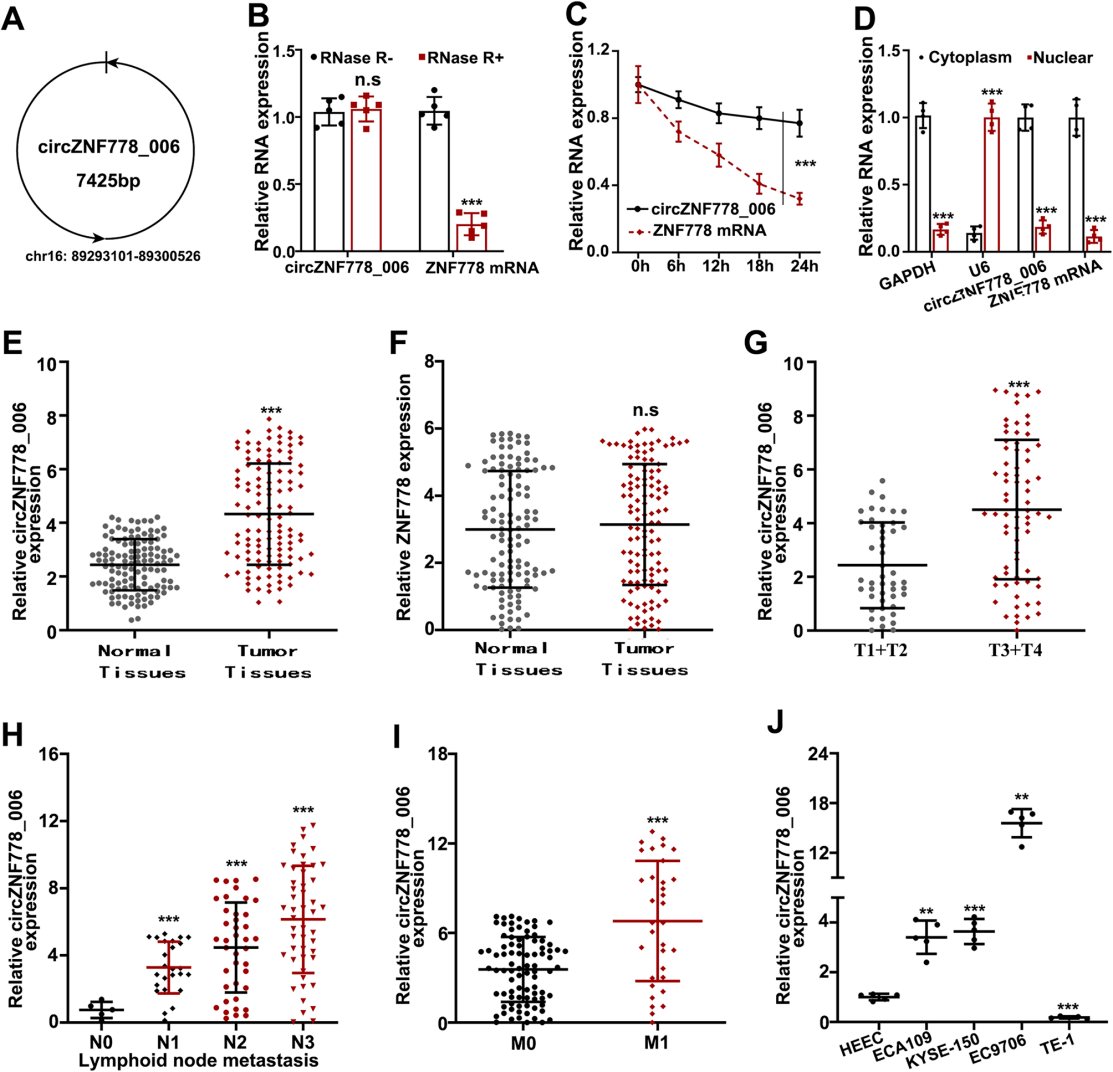
The characters and the expression of circ_ZNF778_006 in ESCC tissues. (**A**) The location and the length of circ_ZNF778_006. (**B**) RT-PCR assay was applied to detect the expression of circ_ZNF778_006 and ZNF778 mRNA treated with RNase R- and R+. ***p < 0.01 versus normal tissues. (**C**) The expression of ZNF778 mRNA was lower than circ_ZNF778_006 treated with actinomycin D at different times. ***p < 0.01 vs. ZNF778 mRNA. (**D**) The expression of GaPDH, circ_ZNF778_006 and ZNF778 mRNA in nuclear was lower than that in cytoplasm. And the expression of U6 was reserved. ***p < 0.01 (**E**) The expression of circ_ZNF778_006 in ESCC tissues (n = 110) was higher than that in normal tissues (n = 110). ***p < 0.01 vs. the normal tissues. (**F**) The expression of ZNF778 mRNA in ESCC tissues (n = 110) was equal to that in normal tissues (n = 110). (**G**) The expression of circ_ZNF778_006 in ESCC tissues of stage T1+2 (n = 45) was lower than that in stage T3+4 (n = 65). ***p < 0.01. (**H**) The expression of circ_ZNF778_006 in ESCC tissues was increased with the Stage increased.***p < 0.01. (**I**) The expression of circ_ZNF778_006 in ESCC tissues of stage M0 (n = 77) was lower than that in M1 (n = 33). ***p<0.01. J The expression of circ_ZNF778_006 in ESCC cell lines (EC9706) was highest, and the expression was reserved in TE-1 cells. **p < 0.05, ***p < 0.011.

The circ_ZNF778_006 is located on the chr16: 89293101–89300526 and the length was 7425 bp based on the circBank data (Fig. 1A). The circ_ZNF778_006 was not affected by the RNase and actinomycin D, but the ZNF778 mRNA was different (Fig. 1B,C). Furthermore, the results showed that the circ_ZNF778_006 mainly is located in the cytoplasm (Fig. 1D). These results showed that the circ_ZNF778_006 is a stable circular RNA in ESCC. The results (relative expression) showed that the expression of circ_ZNF778_006 in ESCC tissues was significantly higher than that in normal tissues (Fig. 1E). But there was no difference in comparison to ZNF778 (Fig. 1F). In the comparison of subgroups, the expression of circ_ZNF778_006 in the high tumor stage was higher than that in the low tumor stage (Fig. 1G–I). These results showed that the expression of circ_ZNF778_006 was positively correlated with the TNM stage. Furthermore, the expression of circ_ZNF778_006 in ESCC cell lines(ECA109, KYSE-150, and EC9706) was higher than that in normal squamous cell lines, but it in the TE-1 cell line was the opposite (Fig. 1J). Thus we chose the EC9706 and TE-1 cell lines for the following study.

### The relationship of the expression of circ_ZNF778_006 and the sensitivity of 5-Fu and Cisplatin

**Figure 2:**
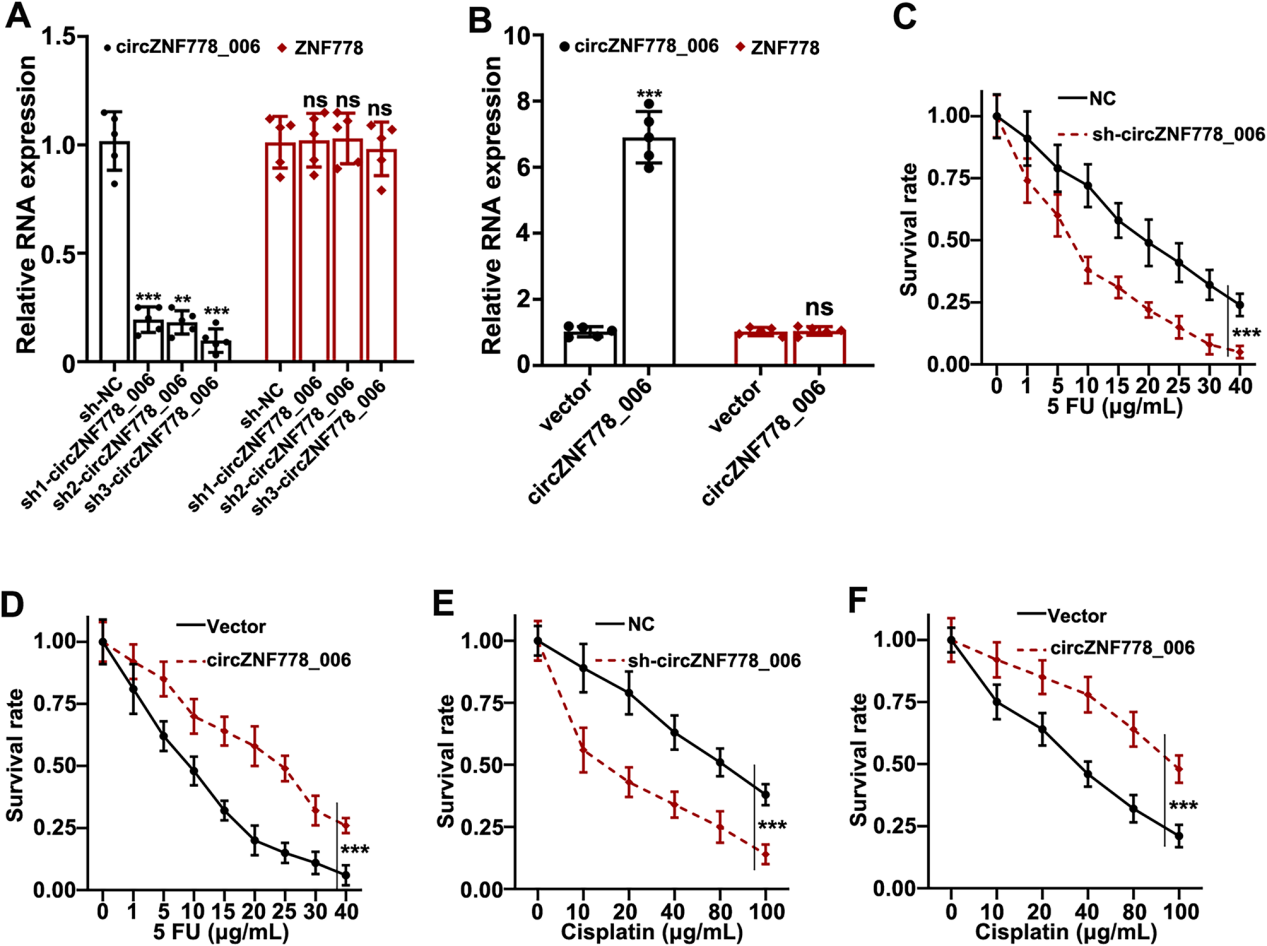
the expression of circ_ZNF778_006 in ESCC cells treated with 5-Fu or Cisplatin. (**A**) RT-PCR assay was applied to detect the expression of circ_ZNF778_006 and ZNF778 mRNA treated with different sh-RNA in the EC9706 cell line. **p < 0.05 vs sh-NC, ***p < 0.01 vs. sh-NC. (**B**) The expression of circ_ZNF778_006 and ZNF778 mRNA transfected with oe-cZNF778_006 in TE-1 cell line. ***p < 0.01 versus. blank vector. (**C**) The survival rate of EC9706 cells transfected with sh-circ_ZNF778-006 was lower than that with sh-NC treated with same levels of 5-Fu, respectively. ***p < 0.01. (**D**) The survival rate of TE-1 cells transfected with circ_ZNF778-006 was higher than that with sh-NC treated with same levels of 5-Fu, respectively. ***p < 0.01. (**E**) The survival rate of EC9706 cells transfected with sh-circ_ZNF778-006 was lower than that with sh-NC treated with same levels of Cisplatin, respectively. ***p < 0.01. (**F**) The survival rate of TE-1 cells transfected with circ_ZNF778-006 was higher than that with empty vector treated with same levels of Cisplatin, respectively. ***p < 0.01.

We constructed different sh-RNA and oe-cZNF778_006 vectors to identify the relationship of circ_ZNF778_006 and 5-Fu and Cisplatin. Different shRNAs could inhibit the expression of circ_ZNF778_006 in EC9706 cells, but the expression of ZNF778 was not affected. And different oe-c ZNF778_006 vectors could increase the expression of circ_ZNF778_006 in TE-1 cells, but the expression of ZNF778 was not affected (Fig. 2A,B). The survival rate of EC9706 cells decreased with the increase of the concentration of 5-Fu, gradually, but the rate in cells after treatment with sh-RNA was lower than that with sh-NC at the same time point (Fig. 2C). And the results were opposite in TE-1 cells after treatment with oe-c ZNF778_006 (Fig. 2D). The results in cells treated with Cisplatin were the same as those treated with 5-Fu (Fig. 2E,F). These results showed that the expression of circ_ZNF778_006 can affect the sensitivity of ESCC cells to chemotherapy.

### Circ_ZNF778_006 deficiency suppressed proliferation, migration, invasion of ESCC cells

**Figure 3:**
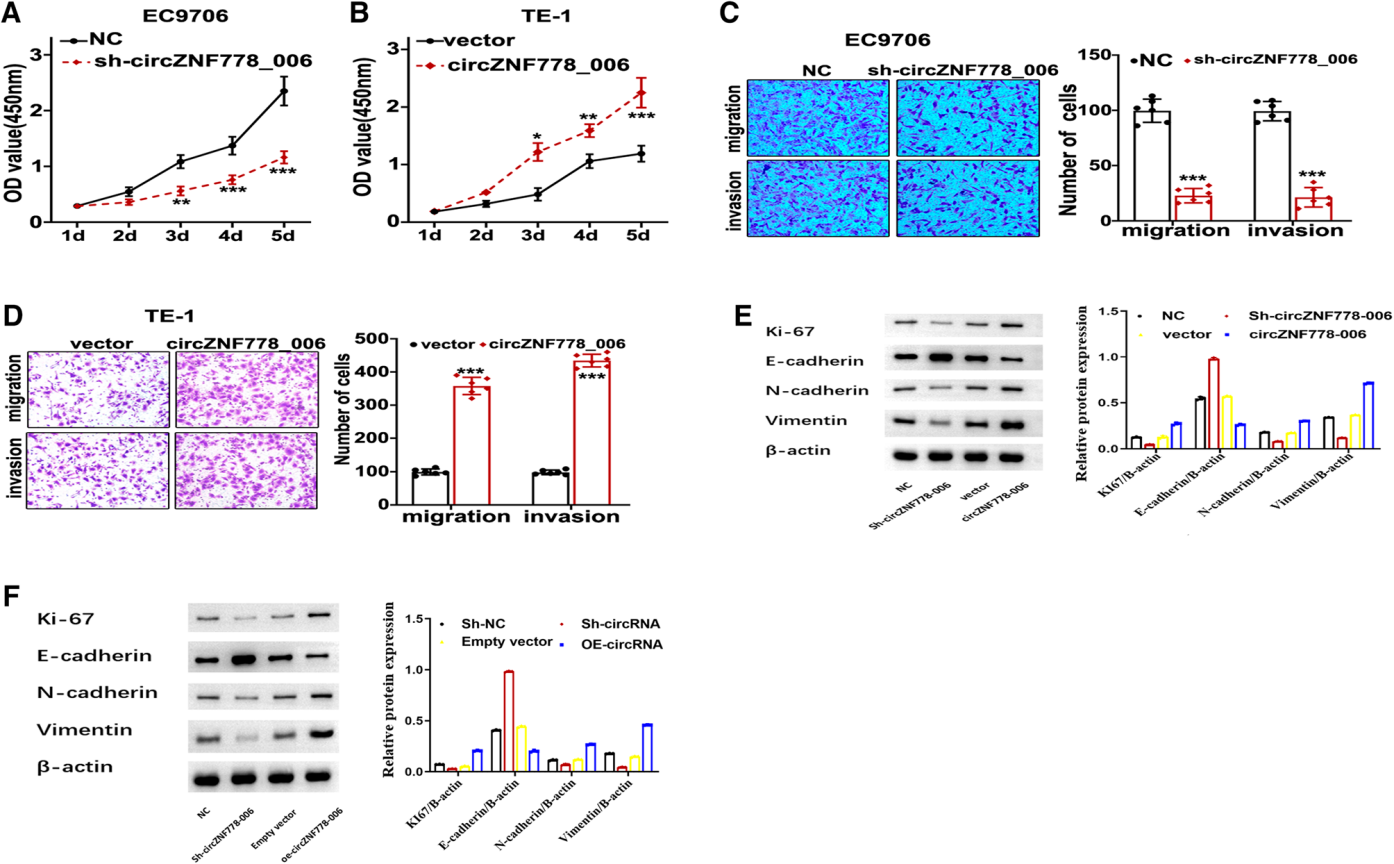
circ_ZNF778_006 deficiency suppressed proliferation, migration, invasion of ESCC cells. (**A**) The proliferation of EC9706 cells treated with sh-RNA was lower than that with sh-NC as the increasing time. **p < 0.05 vs sh-NC, ***p < 0.01 vs. sh-NC. (**B**) The proliferation of TE-1 cells treated with oe-cZNF778_006 was higher than that with empty vector as the increasing time. **p < 0.05 vs blank vector, ***p < 0.01 vs. blank vector. (**C**) The invasion and migration of EC9706 cells treated with sh-RNA was lower than that with sh-NC. ***p < 0.01 vs. sh-NC. (**D**) The invasion and migration of TE-1 cells treated with oe-cZNF778_006 was higher than that with empty vector. ***p < 0.01 vs. blank vector. (**E**) The expression of Ki-67, N-cadherin, and Vimentin mRNA in EC9706 cells treated with sh-RNA was highest in four groups, respectively. But the expression of E-cadherin was lowest. (**F**) The expression of Ki-67, E-cadherin, N-cadherin, and Vimentin mRNA in TE-1 cells treated with oe-circ_ZNF778_006 was highest in four groups, respectively. But the expression of E-cadherin was lowest.

To identify the role of circ_ZNF778_006 in the characteristics of ESCC cells, the EC9706 cells were transfected with sh-RNA, and the TE-1 cells were transfected with oe-RNA, respectively (Fig. 3A,B). After transfection, we could found that the migration and invasion of EC9706 cells were lower than that of normal cells, but the results were opposite in TE-1 cells transfected with oe-RNA (Fig. 3C,D). The results showed that the deficiency of circ_ZNF778_006 could suppress the proliferation, migration, invasion of ESCC cells. Furthermore, to explore the impact of circ_ZNF778_006 in the EMT process of ESCC cells, we analyzed the relationship between circ_ZNF778_006 and the expression of Ki-67, E-cadherin, N-cadherin, and vimentin. After transfection with sh-RNA, the expression of Ki-67, N-cadherin, and vimentin in EC9706 cells was lower than that in normal tumor cells, except for E-cadherin (Fig. 3E). But the results were opposite in TE-1 cells transfected with oe-RNA (Fig. 3F). The results showed that the circ_ZNF778_006 could affect the EMT process of ESCC cells.

### Circ_ZNF778_006 interacted with miR-18b-5p

**Figure 4:**
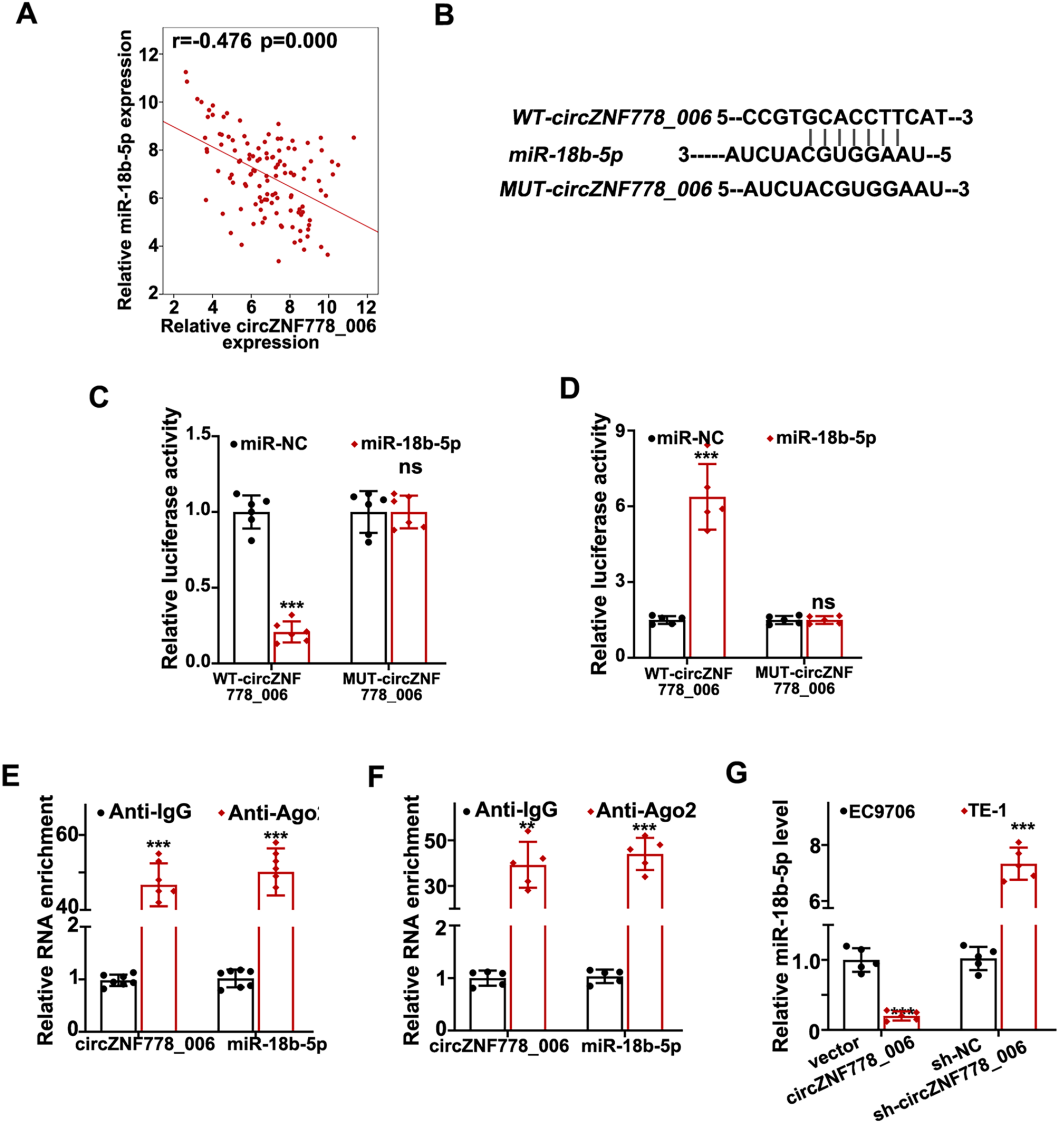
The relationship of circ_ZNF778_006 with miR-18b-5p. (**A**) The relationship of circ_ZNF778_006 with miR-18b-5p was the most obvious analyzed (r = 0.476, *p* = 0). (**B**) The binding sites between WT—circ_ZNF778_006 and miR-18b-5p were predicted based on the Starbase software analysis. (**C**) The luciferase activity in EC9706 cells transfected with WT-circ_ZNF778_006 and miR-18b-5p mimics was lowest. ***p < 0.01. (**D**) The luciferase activity in TE-1 cells transfected with WT-circ_ZNF778_006 and miR-18b-5p mimics was highest. ***p < 0.0. **E** The relationship between circ_ZNF778_006 and miR-18b-5p was verified based on the Ago-RIP analysis in EC9706 cells. ***p < 0.01 vs. miR-NC. (**F**) The relationship between circ_ZNF778_006 and miR-18b-5p was verified based on the Ago-RIP analysis in TE-1 cells. ***p < 0.01 vs. miR-NC. (**G**) The expression of miR-18b-5p after treated with oe-cZNF778_006 and sh-RNA in EC9706 and TE-1 cells. ***p < 0.01 vs. EC9706 cells.

As we know, the circRNAs could exert as the sponge of miRNAs^14^. So we predict the interaction of circRNAs of miRNAs based on the Starbase software data. The results showed that the circ_ZNF778_006 was significantly correlated with mir-18b-5p (Fig. 4A). And miR-18b-5p was found to possess some complimentary sites with circ_ZNF778_006 (Fig. 4B). The luciferase activity in EC9706 cells transfected circ_ZNF778_006 WT and miR-18b-5p mimic was greatly reduced compared with that in cells transfected circRNA WT and miR-NC based on the dual-luciferase reporter results (Fig. 4C). But the results were the opposite in TE-1 cells (Fig. 4D). The next results showed that the circ_ZNF778_006 is bound with miR-18b-5p in EC9706 and TE-1 cells based on the Ago2-RIP analysis (Fig. 4E,F). And the RT-PCR results indicated that the miR-18b-5p level was greatly reduced in TE-1 cells transfected with oe-circ_ZNF778_006 compared with that in EC9706 cells transfected with circRNA-NC, whereas the miR-18b-5p level was greatly increased in TE-1 cells transfected with sh-circ_ZNF778_006 compared with that in EC9706 cells transfected with shRNA-NC (Fig. 4G). Overall, all the results showed that the expression of circ_ZNF778_006 was significantly negatively correlated with mir-18b-5p.

### miR-18b-5p interacted with HIF-1α

**Figure 5:**
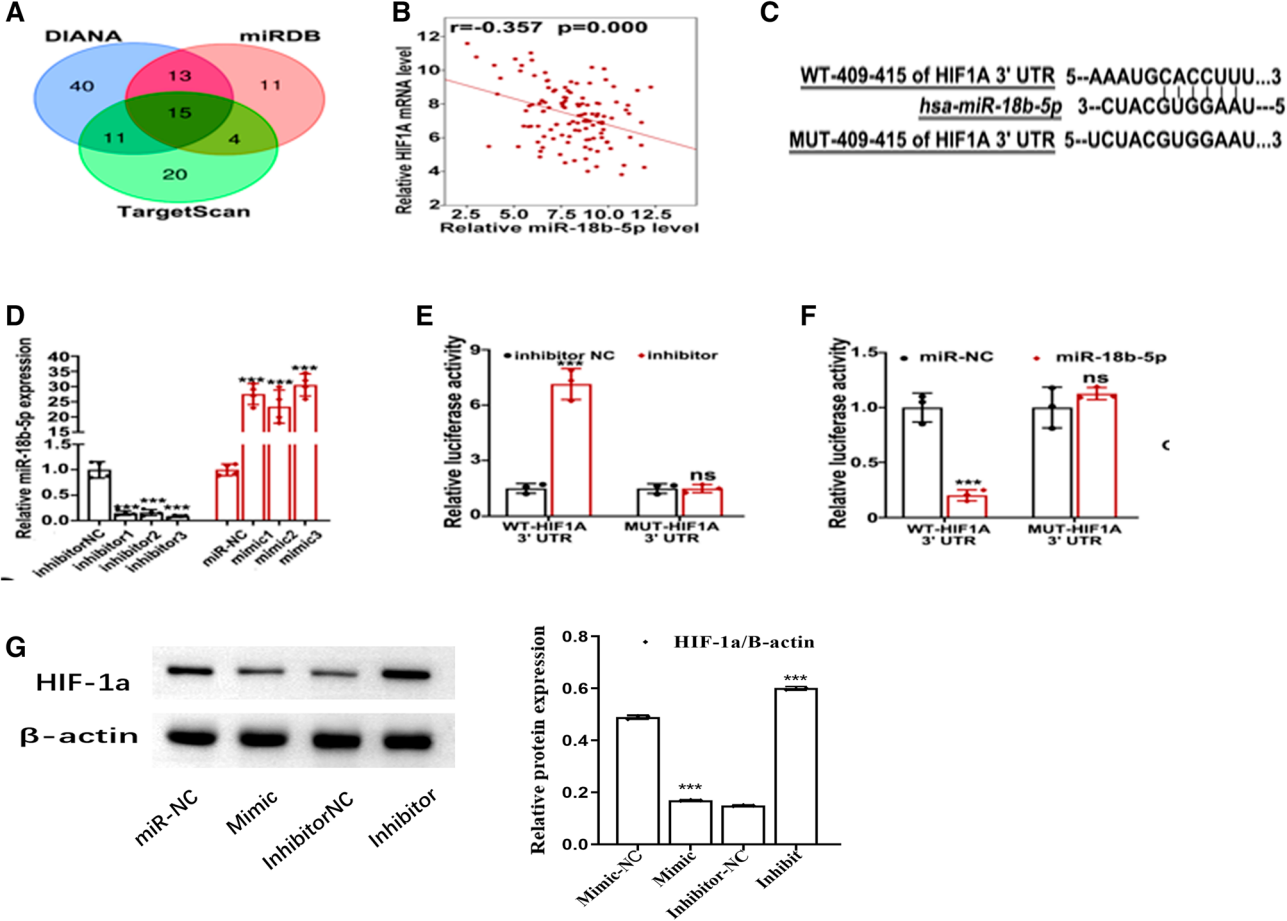
The relationship of miR-18b-5p with HIF-1α. (**A**) The miRNA numbers related to circ _ZNF778_006 were 15 based on the bioinformatics analysis(DIANA, miRDB and TargetScan). B The relationship of circ_ZNF778_006 with miR-18b-5p was the most obvious analyzed (r = 0.357, *p* = 0). (**A**) the target genes were predicted based on the DIANA, miRDB, and TargetScan analysis. (**C**) The binding sites between WT- HIF-1α and miR-18b-5p were predicted based on the Starbase software analysis. (**D**) The expression of miR-18b-5p treated with any different miR-inhibitors NC was lower than that treated with NC. And the expression was reserved treated with different miR-mimics. ***p < 0.01. (**E**) The luciferase activity in EC9706 cells transfected with WT-HIF1A and miR-18b-5p inhibitor was highest. ***p < 0.01. (**F**) The luciferase activity in TE-1 cells transfected with WT-HIF1A and miR-18b-5p mimics was lowest. ***p < 0.01 G The expression of HIF-1A was highest treated with miR-18b-5p inhibitor. ***p < 0.01.

To explore the molecular mechanism of miR-18b-5p, the DIANA, miRDB, and TargetScan analysis was utilized to seek the potential target of miR-18b-5p, which showed that there are 15 potential target genes of miR-18b-5p (Fig. 5A). And the HIF-1α was the significant target gene of miR-18b-5p based on the bioinformatics analysis data (Fig. 5B). The data exhibited that there were some complementary sites between miR-18b-5p and HIF-1α (Fig. 5C). The expression of miR-18b-5p was greatly reduced in EC9706 cells after treatment with different miR-inhibitors compared with that in cells after treatment inhibitors-NC, but the expression of miR-18b-5p was all greatly increased in TE-1 cells after treatment miR-mimics compared that in cells after treatment mimics-NC (Fig. 5D). The luciferase activity in EC9706 cells transfected HIF-1α WT and miR-18b-5p inhibitors were greatly increased compared with that in cells transfected HIF-1α WT and miR-18b-5p inhibitors based on the dual-luciferase reporter results (Fig. 5E). But the results were the opposite in TE-1 cells (Fig. 5F). The expression of HIF-1α protein and HIF-1α mRNA was greatly reduced in EC9706 cells transfected miR-mimics compared with that in cells after treatment miR-NC, but the expression of HIF-1α was all greatly increased in TE-1 cells after treatment miR-inhibitor compared that in cells after treatment inhibitor-NC (Fig. 5G). Overall, the results showed that the HIF-1α was the target gene of miR-18b-5p, and the expression of mir-18b-5p was significantly negatively correlated with HIF-1α.

### The roles of circ_ZNF778_006/miR-18b-5p/HIF-1α axis in cancer progression of ESCC cells

**Figure 6:**
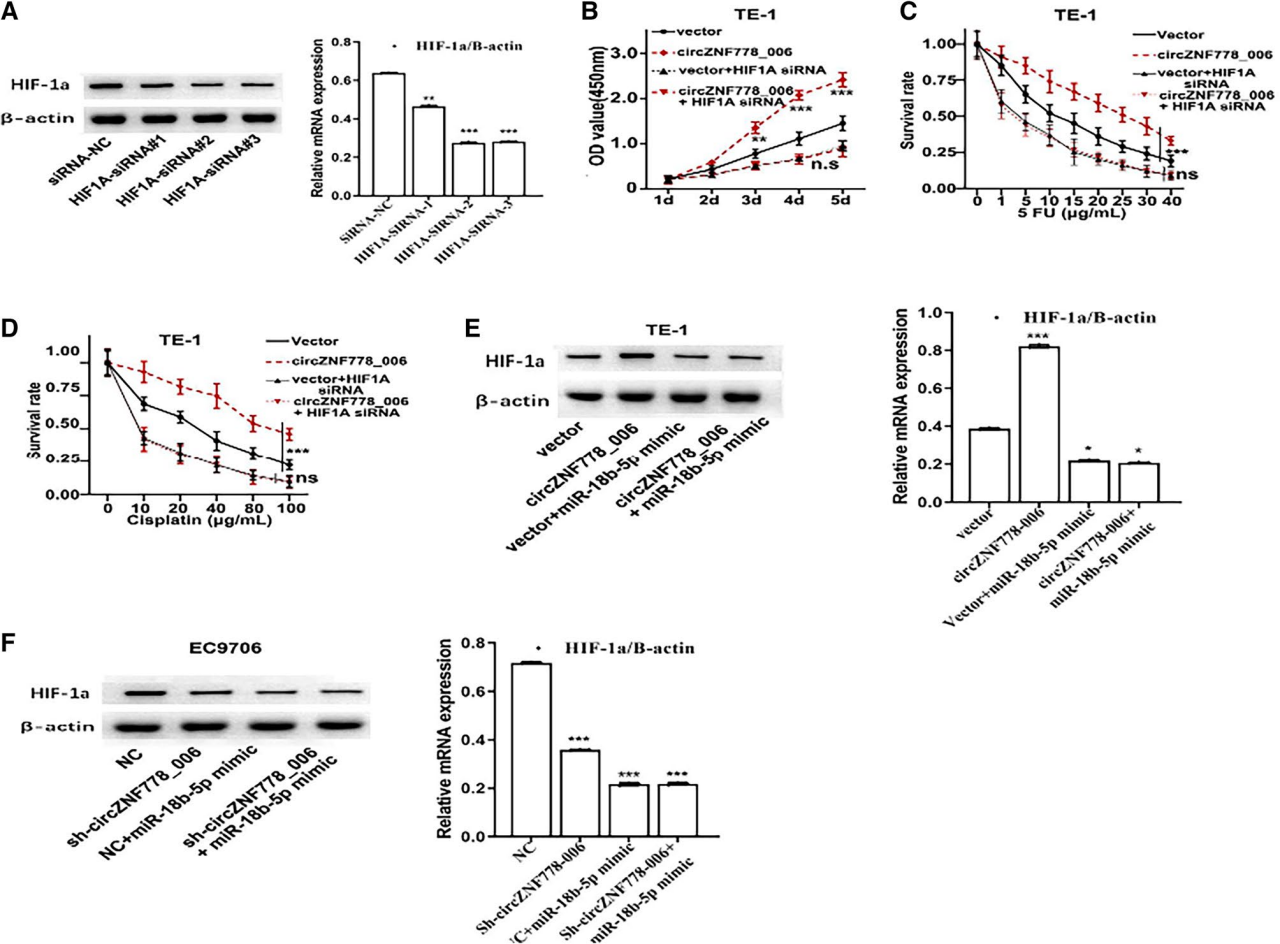
circ_ZNF778_006 promotes HIF-1α by regulating miR-18b-5p. (**A)** The expression of HIF-1α transfected any different HIF-1α-siRNA was lower than that with NC. **p < 0.05, ***p < 0.01. (**B**) The proliferation of TE-1 cells transfected with circ_ZNF778_006 and HIF-1A siRNA was highest in four groups at the same time. **p < 0.05, ***p < 0.01. (**C**) The survival rate of TE-1 cells transfected with circ_ZNF778_006 and HIF-1A siRNA was highest in four groups at the same 5-Fu level. ***p < 0.01. (**D**) The survival rate of TE-1 cells transfected with circ_ZNF778_006 and HIF-1A siRNA was highest in four groups at the same Cisplatin level. ***p < 0.01. (**E**) The expression of HIF-1α in TE-1 cells transfected with circ_ZNF778_006 was highest. *p < 0.05. (**F**) The expression of HIF-1α in EC9706 cells transfected with sh-circ_ZNF778_006 and miR-18b-5p mimic was lowest. ***p < 0.01.

To explore the roles of circ_ZNF778_006/ miR-18b-5p/HIF-1α axis in the cancer progression of ESCC cells, first we constructed TE-1 cells with HIF-1α-siRNAs by transfecting with different HIF-1α siRNAs (Fig. 6A). And the AKP assay results showed that the over-expression of circ_ZNF778_006 could promote the proliferation of TE-1 cells, but the HIF-1α suppression could abrogate circ_ZNF778_006-mediated proliferation in TE-1 cells (Fig. 6B). The drug sensitivity results showed that the over-expression of circ_ZNF778_006 could promote the resistance of 5-Fu and cisplatin in TE-1 cells, but the HIF-1α suppression could reduce the circ_ZNF778_006-mediated resistance in TE-cells (Fig. 6C,D). So we could find that the expression of circ_ZNF778_006 promoted the proliferation and drug resistance of TE-1 cells by regulating the expression of HIF-1α. To explore the effect of miR-18b-5p in circ_ZNF778_006/miR-18b-5p/HIF-1α axis, we constructed the TE-1 cells transfected none/oe-circRNA/miR-mimic/ oe-circRNA and miR-mimic, respectively. Meanwhile, the EC9706 cells were constructed by transfection with none/sh-circRNA/miR-mimic/ sh-circRNA and miR-mimic, respectively. The Wb and RT-PCR results showed that the over-expression of circ_ZNF778_006 could promote the expression of HIF-1α protein and HIF-1α mRNA in TE-1 cells, but the miR-18b-5p over-expression could abrogate circ_ZNF778_006-mediated over-expression of HIF-1α in TE-1 cells (Fig. 6E). And the results were opposite in EC9706 cells (Fig. 6F). The above results showed that the circ_ZNF778_006 could promote the expression of HIF-1α by suppressing miR-18b-5p.

### Circ_ ZNF778_006up-regulation inhibited ESCC cell proliferation in vivo

**Figure 7:**
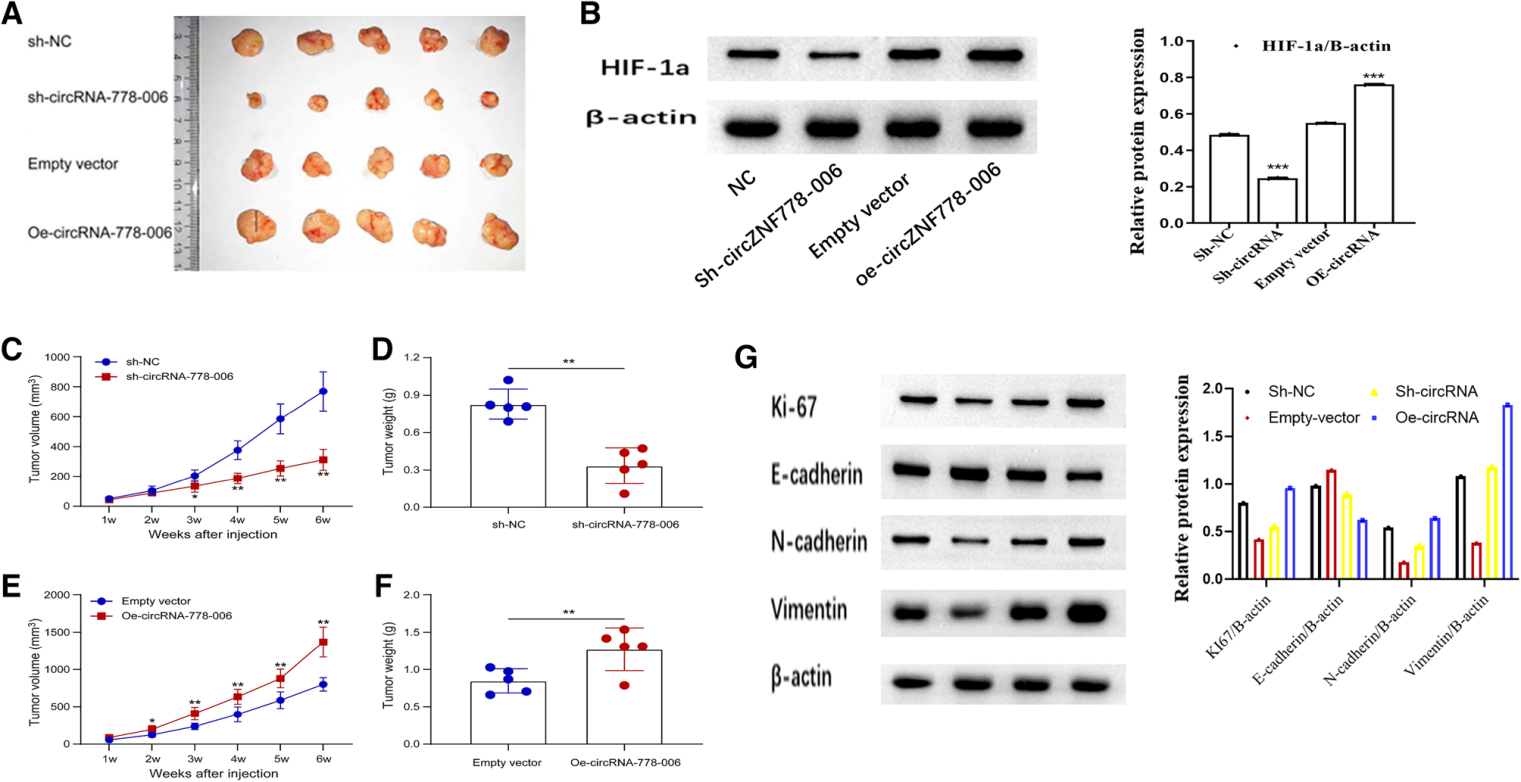
circ_ZNF778_006 can promoted ESCC cell proliferation in vivo. (**A**) The volume of xenograft tumor in nude mice. (**B**) The expression of HIF-1a was highest in xenograft tumor of nude mice treated with oe-circRNA778_006 was largest. ***p < 0.01. (**C**) The subcutaneous tumor volume in the xenograft tumor of nude mice treated with sh-circRNA778_006 was lower than that with sh-NC. *p < 0.05, **p < 0.01. (**D**) The tumor weight in the xenograft tumor of nude mice treated with sh-circRNA778_006 was lower than that with sh-NC. **p < 0.01. (**E**) The subcutaneous tumor volume in the xenograft tumor of nude mice treated with oe-circRNA778_006 was higher than that with sh-NC. *p < 0.05, **p < 0.01. (**F**) The tumor weight in the xenograft tumor of nude mice treated with oe-circRNA778_006 was higher than that with sh-NC. **p < 0.01. (**G**) The expression of Ki-67, N-cadherin, and Vimentin mRNA in rats treated with oe-circRNA was highest in four groups, respectively. But the expression of E-cadherin was lowest.

Finally, the ECA-109 tumor mice model was established for evaluating the influence of circ_ZNF778_006 levels in vivo. ECA-109 cells transfected with or without sh-circ_ZNF778_006 were given into the dorsal side of the nude mice via subcutaneous injection. Figure 7A is a visual representation of tumor morphology in all groups (Fig. 7A). As observed, The expression of HIF-1a was highest in xenograft tumor of nude mice treated with oe-circRNA778_006 was largest (Fig. 7B). And the mice in the sh-circ_ZNF778_006 transfected group had remarkably decreased tumor volume and weight than the sh-NC treatment group (Fig. 7C,D). Correspondingly, compared with the oe-circ_ZNF778_006 treatment group and the empty vector group, the volume and weight of the overexpression group increased significantly (Fig. 7E,F). Furthermore, the expression of Ki-67, N-cadherin, and Vimentin mRNA in rats treated with oe-circRNA was highest in four groups, respectively. But the expression of E-cadherin was lowest (Fig. 7G).

## Discussion

Despite advances in clinical comprehensive treatment, the prognosis of ESCC remains poor^2^. So it is of great significance to find the ESCC pathogenesis that can improve its prognosis. CircRNAs are closely related to many tumors^4^, including ESCC^5^. Here, our results revealed that circ_ZNF778_006 acts as a cancer-promoting factor in the development of ESCC.

Previous studies have revealed that many circular RNAs are tumor-promoting factors in ESCC. Chen et al. found that circNTRK2 expression was significantly increased in ESCC, which promote cell proliferation and invasion^6^. And Zhang et al. also found that Circ_0014715 could promote the cell proliferation and inhibits apoptosis in ESCC^15^.We found that the expression of circ_ZNF778_006 was increased in ESCC compared with the normal tissues. And it also was positively correlated with the pathological T, N, and M stages. Previous studies have reported that circRNAs can affect the sensitivity of ESCC to chemotherapeutic drugs. Qu et al.^16^ found that circ_0006168 can enhance the resistance of ESCC to paclitaxel. We also found that overexpression of circ_ZNF778_006 can inhibit the sensitivity of ESCC to 5Fu and cisplatin. Furthermore, the circ_ZNF778_006 can promote the proliferation, invasion, migration, and EMT process of ESCC cells in functional experiments. These results suggest that circ_ZNF778_006 may play a role in promoting the progression of ESCC.

Some studies have confirmed that circRNAs are involved in the tumor biological process by virtue of acting as ceRNA or miRNA sponge^14,17^. The function of miRNA in malignant tumors has been widely studied, including ESCC^7,18^. But the role of miR-18b-5p in ESCC has still not been reported. Xu et al. found that exosome transfer of annular RNAFBXW7 can improve chemical resistance to oxaliplatin in colorectal cancer by sponging miR-18b-5p^9^. And our results showed that circ_ZNF778_006 is the molecular binding site of miR-18b-5p in ESCC, also it can inhibit the expression of miR-18b-5p.

HIF-1α is a critical hypoxia adaptive transcription factor for the responses to hypoxia. It plays an important role in many kinds of malignant tumors by activating the expression of many hypoxia-responsive genes^10,11,19^. HIF-1α was also found to be a poor prognostic factor for ESCC^12,20^. And in recent years, the miRNA/HIF-1α axis was comfirmed to be an important regulatory role in malignant tumors. Xia et al.^21^ found that mir-194-5p enhances the sensitivity of non-small cell lung cancer to adriamycin by targeting the inhibition of HIF-1α. In our study, the HIF-1α contains a potential complementary binding site with miR-18b-5p by using bioinformatics analysis. And it also is verified by double luciferase system analysis. In addition, our results comfirmed that the expression of miR-18b-5p is negatively correlated with HIF-1α expression. And silencing of HIF-1α could inhibit the proliferation of TE-1 cells. The other results comfirmed that HIF-1α can inhibit the sensitivity of 5FU and cisplatin via upregulating of circZNF778_006. The circRNA-miRNA-mRNA interacting network played an important role in the development of cancers. Tang et al. found that the circ_0006948 contributes to ESCC progression via regulating the microRNA-3612/LASP1 axis^22^. And Huang et al. found that circ_0006168 can promote the migration, invasion, and proliferation of ESCC cells via miR-516b-5p dependent regulation of XBP1^23^. Our results also found that the circZNF778_006/miR-18b-5p/HIF-1α network can affect the progression of ESCC.

## Conclusion

Our results comfirmed that the circ_ZNF778_006 is positively correlated with the pathological stage in ESCC. Upregulating of Circ_ZNF778_006 could inhibit the sensitivity of 5-Fu and cisplatin. And it could promote the proliferation, invasion, migration, and EMT process of ESCC cells. Furthermore, it could promote the expression of HIF-1α by sponging miR-18b-5p. The circZNF778_006/miR-18b-5p/HIF-1α network can affect the progression of ESCC (Supplementary file).

### Supplementary Information

Supplementary Figures.

Supplementary Figures.

Supplementary Figures.

## Data Availability

All data relevant to the results are included in this manuscript. Further information on the raw data can be obtained upon request from the corresponding author.
