# Supplementary figures and images for: Circ_ZNF778_006 promoted ESCC progression by upregulating HIF-1α expression via sponging miR-18b-5p
Source: Sci Rep. 2023 Nov 8;13:19363. doi: 10.1038/s41598-023-46832-3 (PMC10632521; doi:10.1038/s41598-023-46832-3)

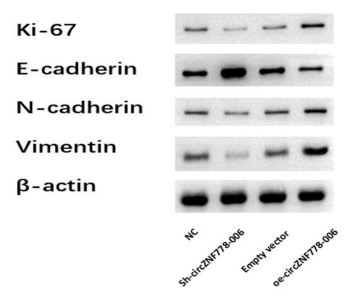

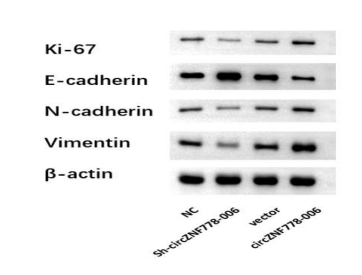


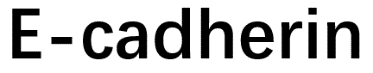

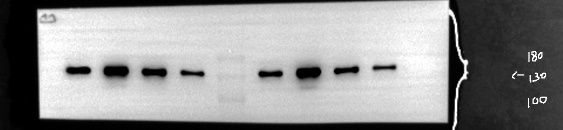

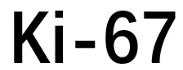

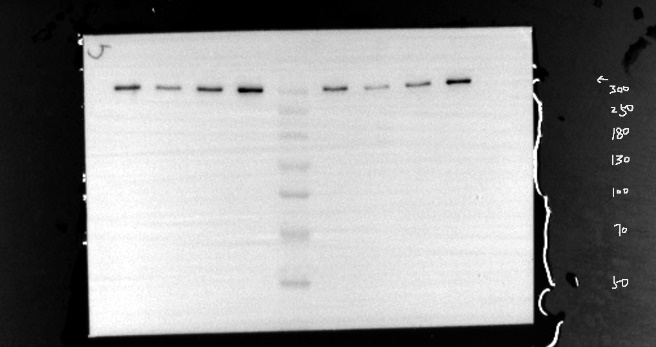


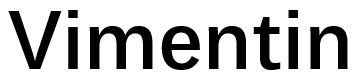

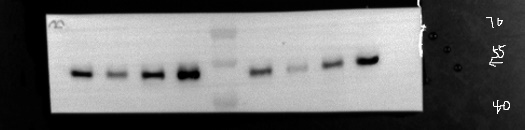

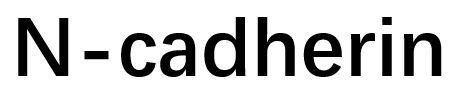

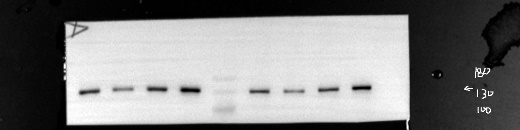


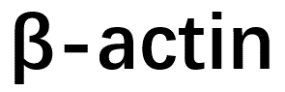

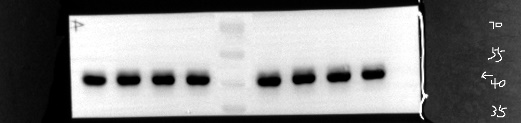


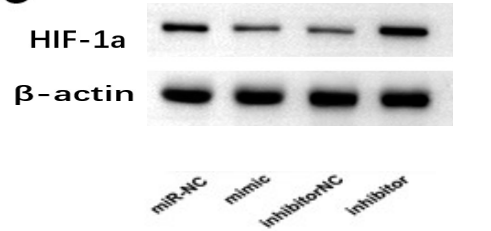

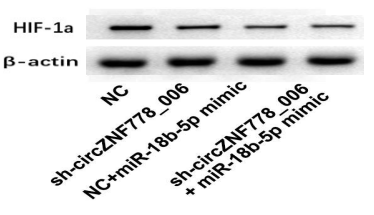


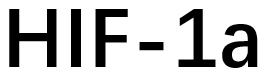

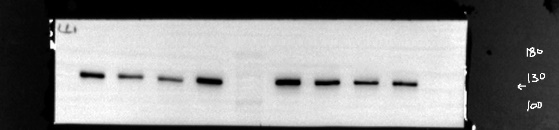


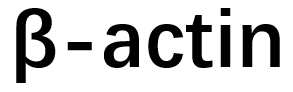

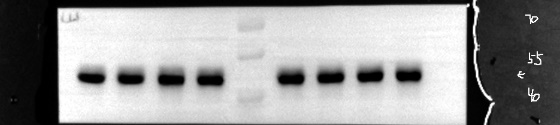


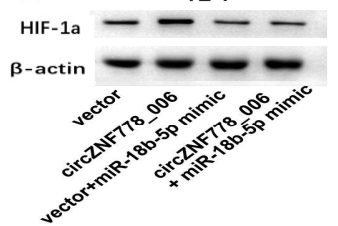

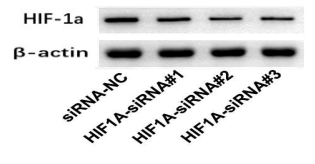


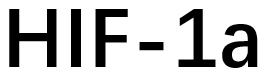

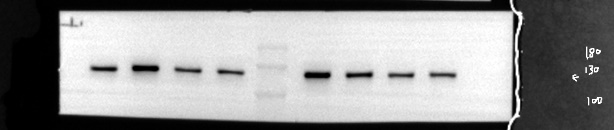


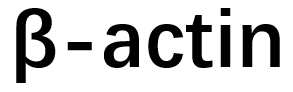

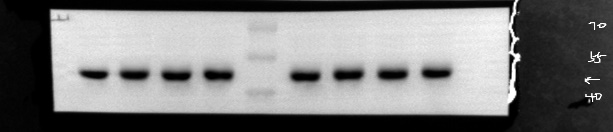


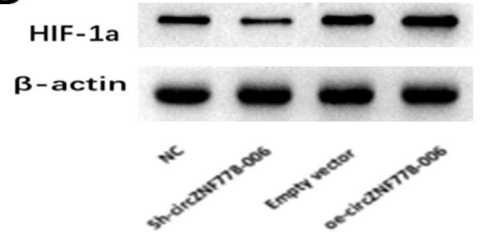


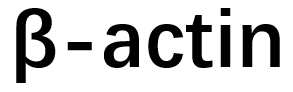

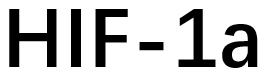

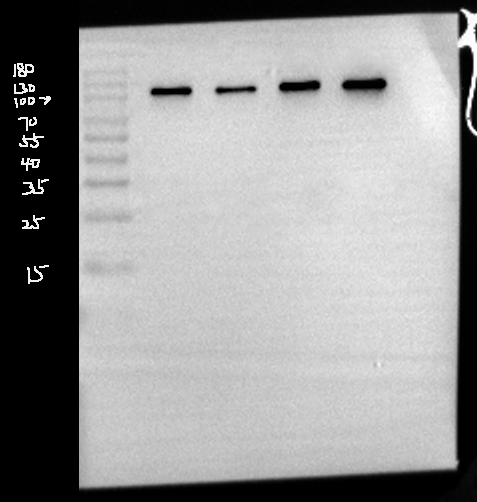

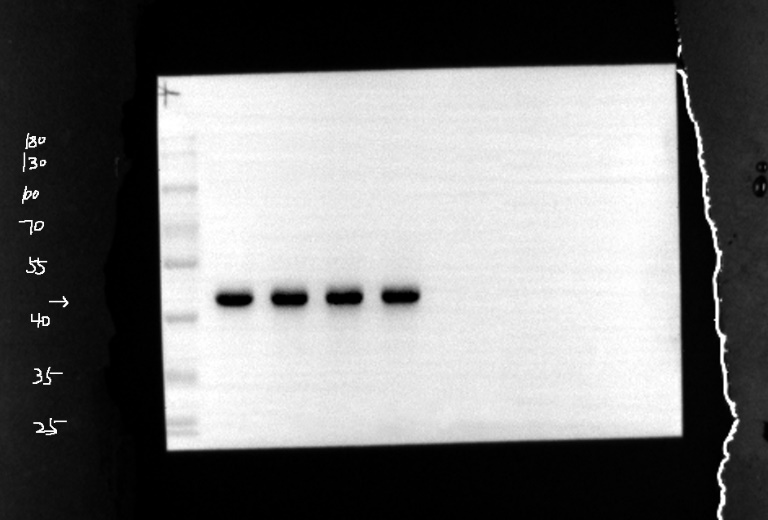


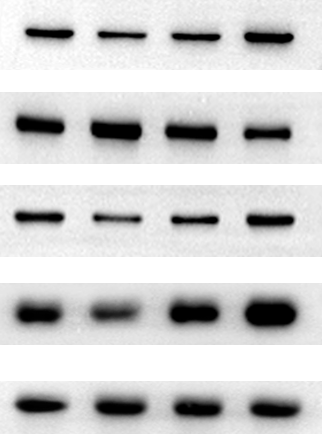

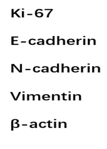
 Fig7E


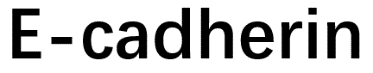

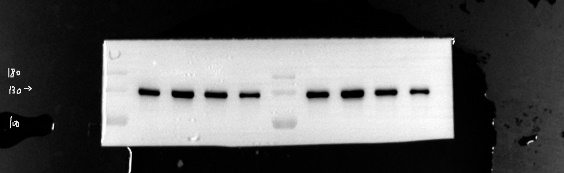

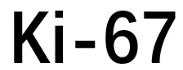

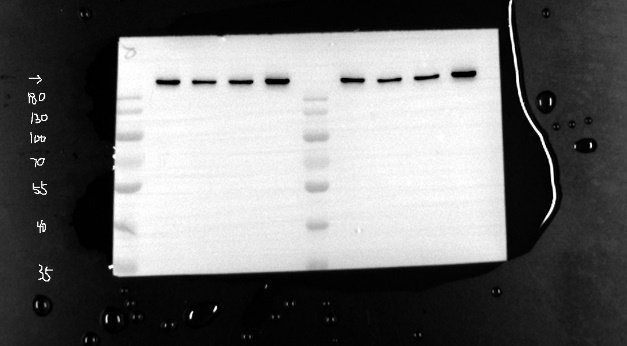


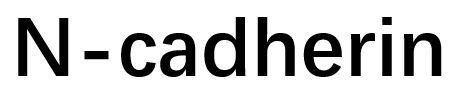

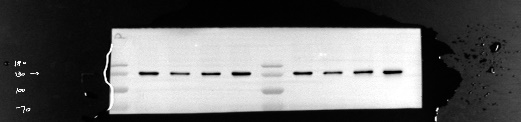

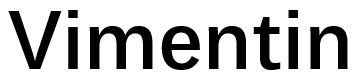

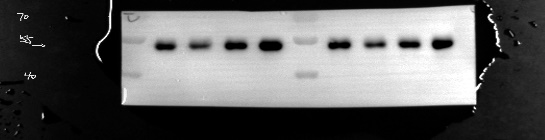


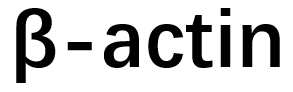

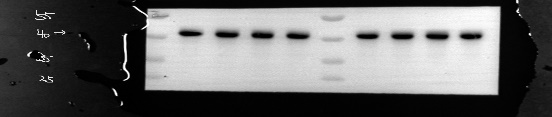

Supplement: Supplementary file 1 — Supplementary Figures. [file 41598_2023_46832_MOESM1_ESM.docx]

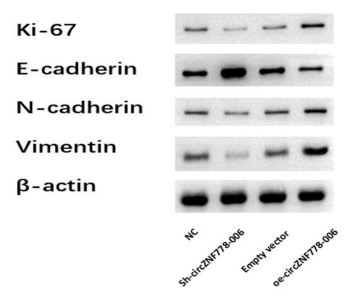

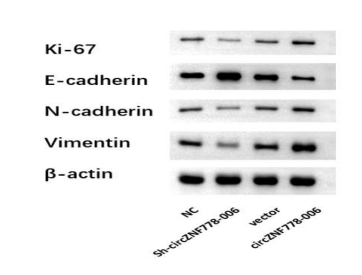


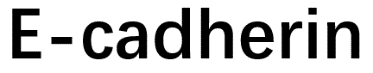

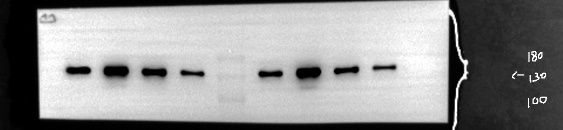

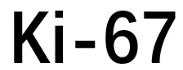

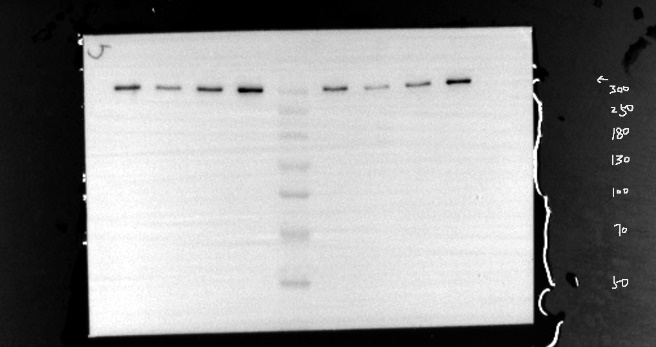


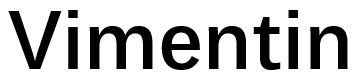

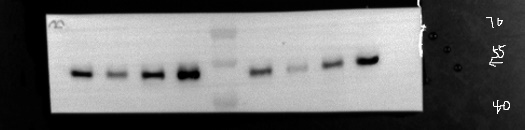

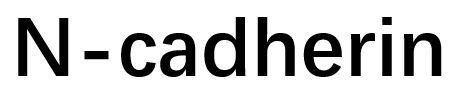

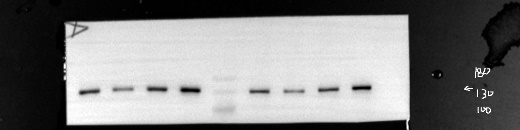


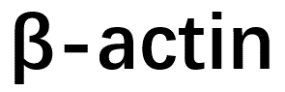

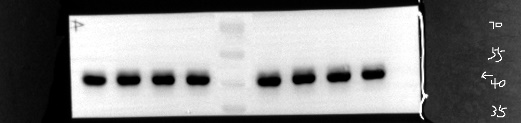


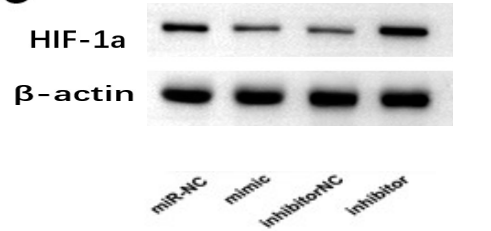

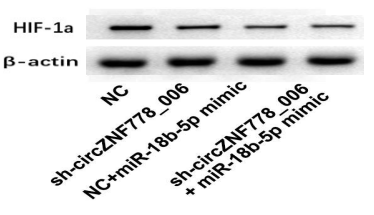


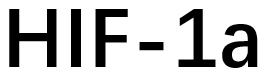

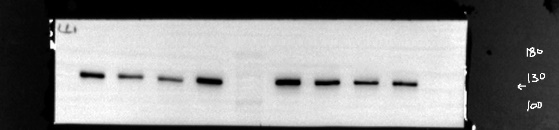


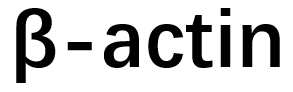

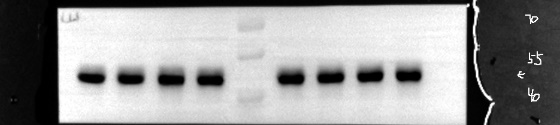


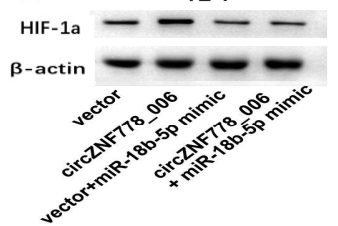

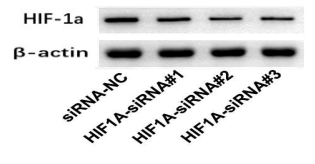


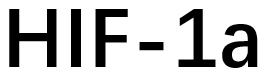

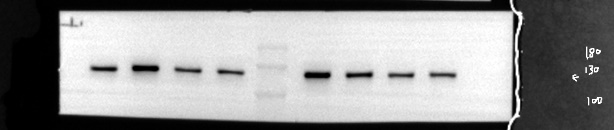


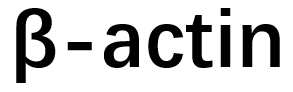

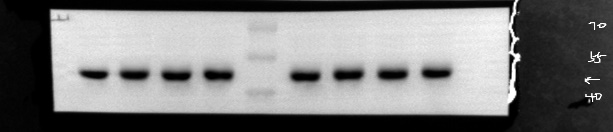


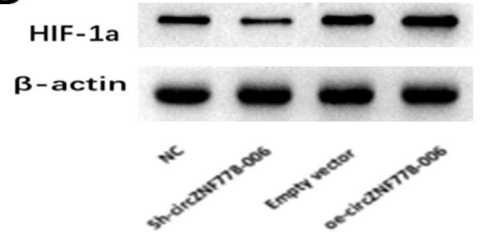


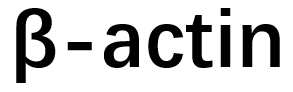

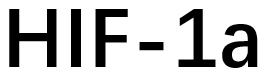

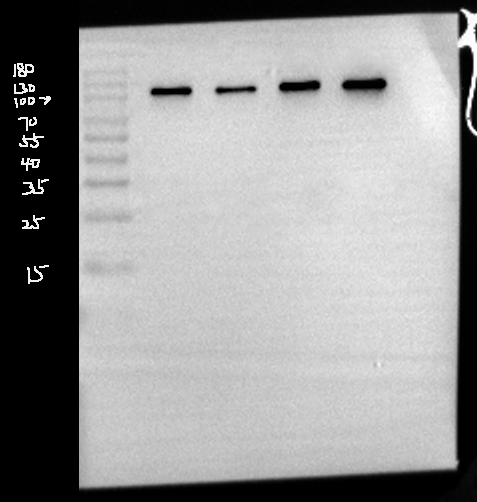

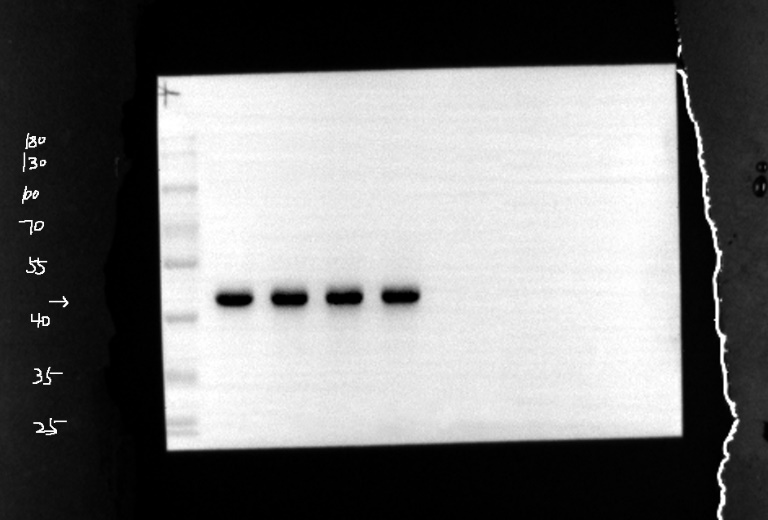

Supplement: Supplementary file 2 — Supplementary Figures. [file 41598_2023_46832_MOESM2_ESM.docx]

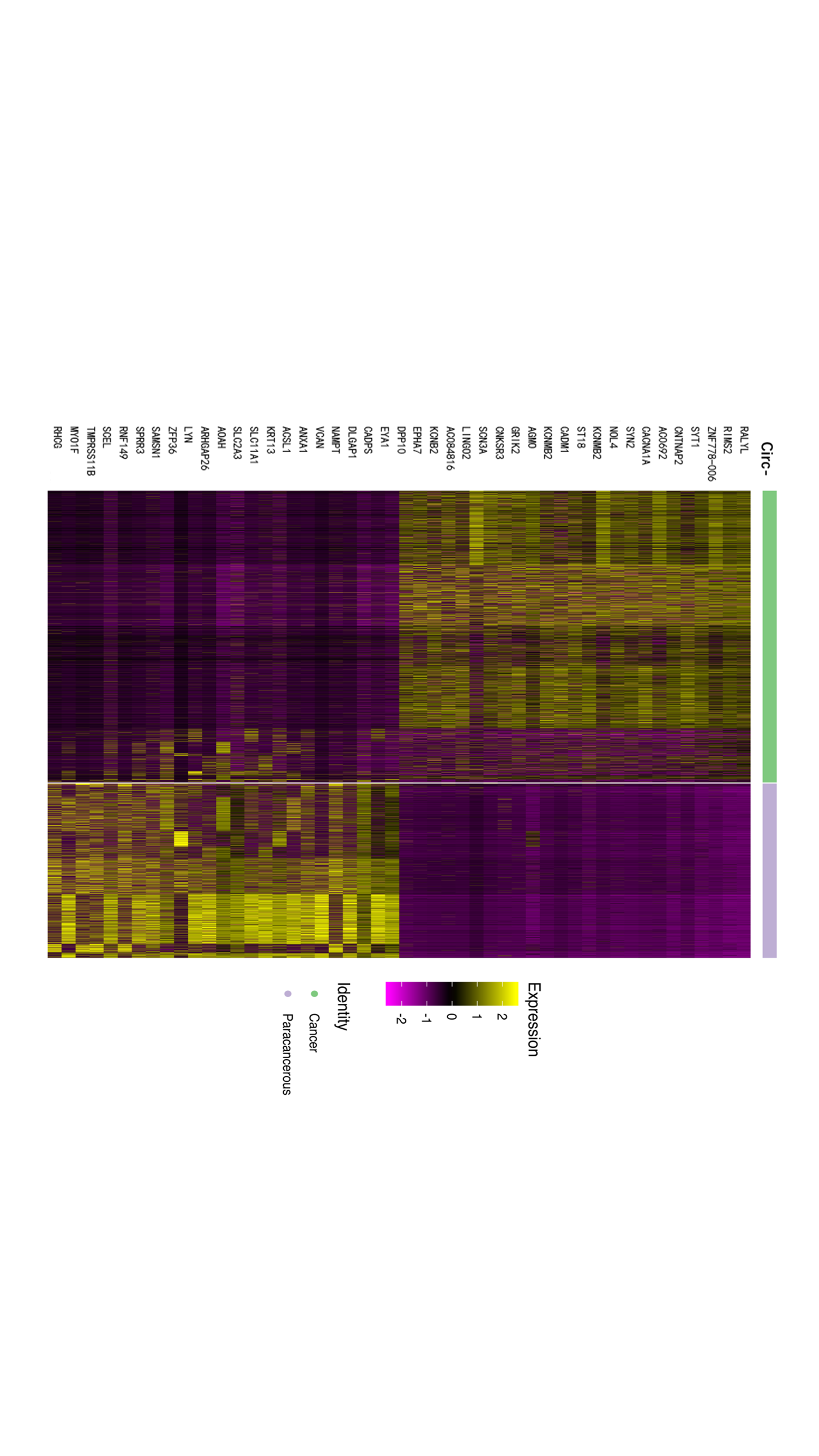

Supplement: Supplementary file 3 — Supplementary Figures. [file 41598_2023_46832_MOESM3_ESM.tif]
